# Smad7 knockdown activates protein kinase RNA-associated eIF2*α* pathway leading to colon cancer cell death

**DOI:** 10.1038/cddis.2017.103

**Published:** 2017-03-16

**Authors:** Veronica De Simone, Gerolamo Bevivino, Silvia Sedda, Roberta Izzo, Federica Laudisi, Vincenzo Dinallo, Eleonora Franzè, Alfredo Colantoni, Angela Ortenzi, Silvia Salvatori, Piero Rossi, Giuseppe S Sica, Massimo C Fantini, Carmine Stolfi, Giovanni Monteleone

**Affiliations:** 1 Department of Systems Medicine, University of ‘Tor Vergata', Rome, Italy; 2 Department of Surgery, University of ‘Tor Vergata', Rome, Italy

## Abstract

Upregulation of Smad7, an inhibitor of transforming growth factor-*β*1 (TGF-*β*1), occurs in sporadic colorectal cancer (CRC) and knockdown of Smad7 inhibits CRC cell growth, a phenomenon that associates with decreased expression of cell division cycle 25 homolog A and arrest of cells in the S phase of the cell cycle. These findings occur in CRC cells unresponsive to TGF-β1, thus suggesting the existence of a Smad7-mediated TGF-β1-independent mechanism that controls CRC cell behavior. Here we show that Smad7 inhibition with a specific Smad7 antisense oligonucleotide upregulates eukaryotic translation initiation factor 2*α* (eIF2*α*) phosphorylation, a transcription factor involved in the regulation of cell cycle arrest and induction of cell death, and induces activating transcription factor 4 (ATF4) and CCAAT/enhancer binding protein homology protein (CHOP), two downstream targets of eIF2*α*. Among the upstream kinases that control eIF2*α* phosphorylation, the serine–threonine protein kinase RNA (PKR), but not general control non-derepressible 2 (GCN2) and protein kinase RNA-like endoplasmic reticulum kinase (PERK), is activated by Smad7 knockdown. PKR silencing abolishes Smad7 antisense-induced eIF2*α* phosphorylation and ATF4/CHOP induction, thereby preventing Smad7 antisense-driven cell death. Smad7 inhibition diminishes interaction of PKR with protein kinase inhibitor p58 (p58^IPK^), a cellular inhibitor of PKR, but does not change the expression and/or activity of other factors involved in the control of PKR activation. These findings delineate a novel mechanism by which Smad7 knockdown promotes CRC cell death.

Colorectal cancer (CRC) is the third most common malignancy and the fourth most common cause of cancer mortality worldwide.^1^ Sporadic CRC accounts for ~70% of all CRC and the development of this neoplasia seems to be influenced by environmental factors^2^ and accumulation of mutations in genes controlling cell growth and survival (e.g. adenomatous polyposis coli, Smad4 and tumor protein 53).^3^ The uncontrolled growth of CRC cells is sustained by immune cells infiltrating the tumor tissue and resident stromal cells.^4, 5^ Moreover, CRC cells overexpress a variety of molecules that act as autocrine factors potentially involved in the early and late events of tumorigenesis. In this context, we have recently shown that CRC cells express high levels of Smad7,^6^ an intracellular protein, which is known for its ability to antagonize transforming growth factor-*β*1 (TGF-*β*1) signaling through multiple mechanisms.^7^ Inhibition of Smad7 with a specific Smad7 antisense oligonucleotide induces CRC cells to block in the S phase of the cell cycle, thus reducing CRC cell growth *in vitro* and in mouse models of sporadic CRC.^6^ Analysis of specific cell cycle checkpoint pathways revealed that Smad7 knockdown in CRC cells leads to inactivation of cyclin-dependent kinase 2, a phenomenon caused by eukaryotic initiation factor 2*α* (eIF2*α*)-mediated cell division cycle 25 homolog A protein downregulation.^6^ Such findings were documented in CRC cell lines unresponsive to TGF-*β*1,^6^ suggesting that Smad7 controls intracellular pathways, which sustain CRC cell growth and survival through a TGF-*β*-independent mechanism.

The aim of the present study was to assess further the basic mechanism by which Smad7 knockdown affects CRC cell behavior and particularly to analyze the factors involved in the hyperphosphorylation/activation of eIF2*α*.

## Results

### Smad7 inhibition activates eIF2*α* pathway

**Figure 1:**
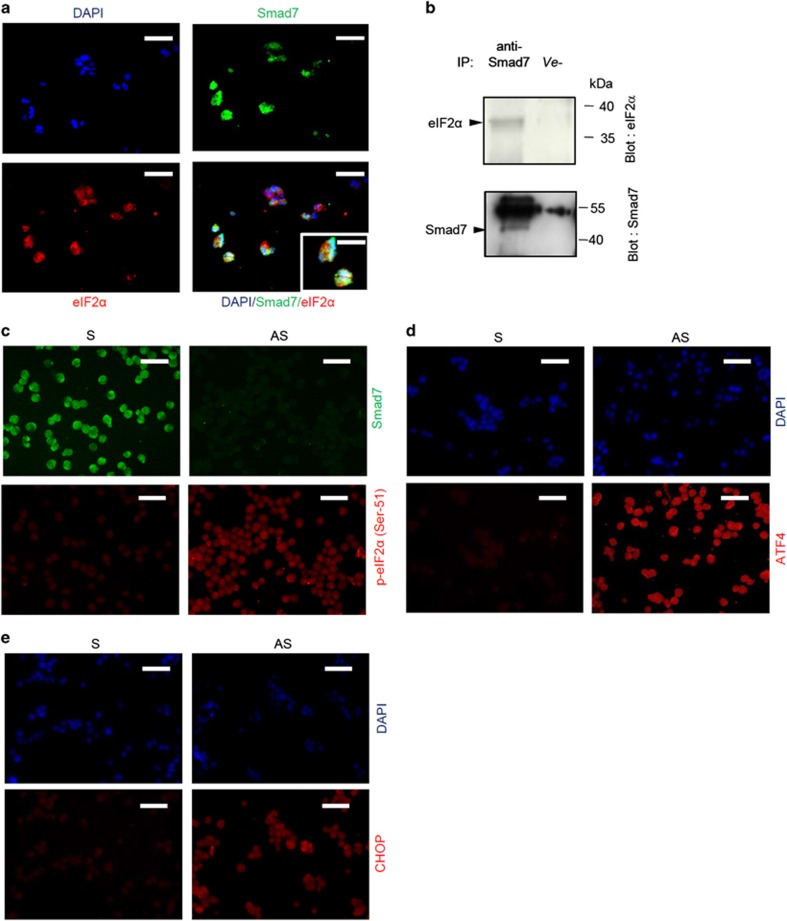
Smad7 colocalizes and interacts with eIF2*α* in colon cancer cells and controls eIF2*α* downstream signaling. (**a**) Representative confocal laser scanning microscopy images showing Smad7 and eIF2*α* colocalization in DLD-1 cell line. Scale bars, 25 *μ*m; scale bar inset, 10 *μ*m. (**b**) Total proteins extracted from DLD-1 cells were immunoprecipitated by an anti-human Smad7 or control isotype (ve−) antibody and then subjected to immunoblotting analysis using eIF2*α* and Smad7 antibodies. One of three representative experiments in which similar results were obtained is shown. (**c**) Representative immunofluorescence pictures of DLD-1 cells showing that Smad7 knockdown enhances eIF2*α* (Ser-51) phosphorylation. Cells were transfected with either Smad7 sense (S) or antisense (AS) oligonucleotide (both used at 2 *μ*g/ml). After 24 h, cells were cultured for further 6 h, fixed and stained as described in (**a**). One of three representative experiments is shown. Scale bars, 20 *μ*m. (**d** and **e**) Representative immunofluorescence images of DLD-1 cells showing an increase of ATF4 and CHOP expression following Smad7 knockdown. Cells were transfected with either Smad7 S or AS (both used at 2 *μ*g/ml). After 24 h, cells were cultured for further 12 h, fixed and stained with DAPI nuclear staining (blue), anti-ATF4 or anti-CHOP and secondary Alexa Fluor 546 antibody (red). One of three representative experiments is shown. Scale bars, 20 *μ*m

To confirm that knockdown of Smad7 leads directly to activation of eIF2*α*, we initially carried out immunofluorescence studies to determine whether these two factors colocalize in DLD-1 and HCT-116 cell lines. EIF2*α* and Smad7 colocalized within the same cells (Figure 1a and Supplementary Figure 1a). Immunoprecipitation of whole-cell extracts from DLD-1 and HCT-116 cells using anti-Smad7 antibody followed by western blotting analysis with an anti-eIF2*α* antibody demonstrated that the endogenous Smad7 interacted with eIF2*α* (Figure 1b and Supplementary Figure 1b). Moreover, cells treated with Smad7 antisense showed higher levels of phosphorylated eIF2*α* than cells treated with Smad7 sense oligonucleotide (Figure 1c and Supplementary Figure 1c).

Phosphorylation of eIF2*α* attenuates mRNA translation of many molecules, but at the same time it induces the activation of several stress-related transcription factors, such as ATF4 and CHOP.^8^ Consistent with the above findings, inhibition of Smad7 was accompanied by induction of ATF4 and CHOP (Figures 1d, e, Supplementary Figures 1d and e).

### Smad7 knockdown determines activation of PKR, a kinase that controls eIF2*α* phosphorylation

**Figure 2:**
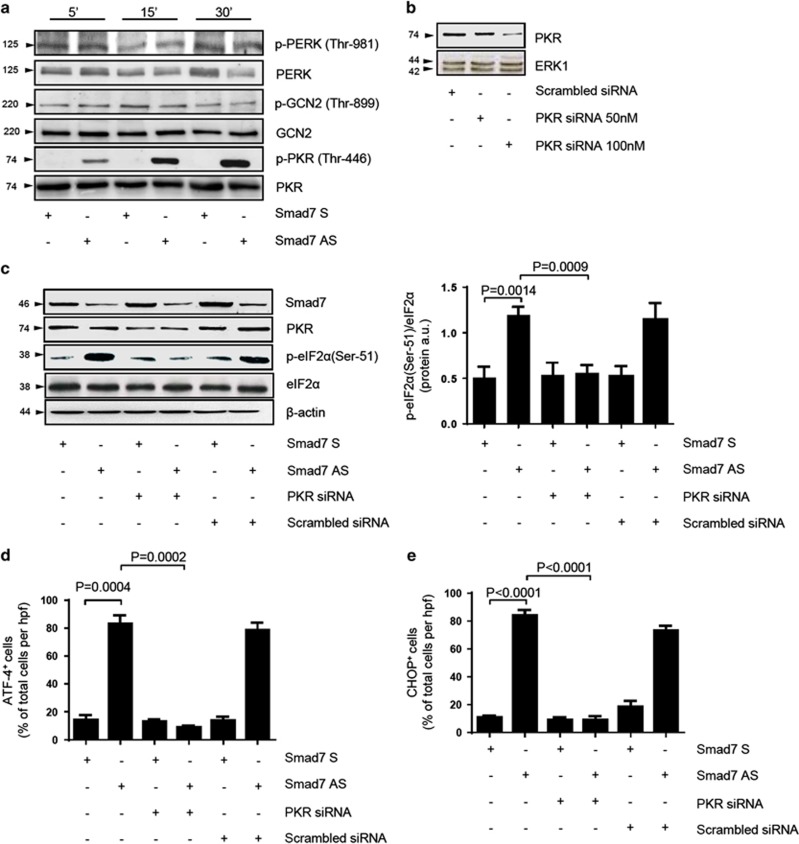
Smad7 antisense (AS)-induced eIF2*α* phosphorylation in DLD-1 cells relies on PKR activation. (**a**) DLD-1 cells were transfected with either Smad7 sense (S) or AS oligonucleotide (both used at 2 *μ*g/ml). After 24 h, cells were washed with PBS and cultured for 5, 15 and 30 min. p-PERK (Thr-981), p-GCN2 (Thr-899) and p-PKR (Thr-446) expression was assessed by western blotting. One of three representative experiments is shown. (**b**) Representative western blots for PKR in extracts of DLD-1 cells transfected with either scrambled-siRNA (100 nM) or increasing doses (50–100 nM) of PKR-siRNA for 24 h. ERK1 was used as a loading control. One of three representative experiments is shown. (**c**) Left panel: Cells were transfected with either Smad7 S or AS oligonucleotide (both used at 2 *μ*g/ml). After 24 h, cells were incubated with either PKR-siRNA or scrambled-siRNA (both used at 100 nM) for further 24 h and then washed with PBS and cultured for additional 6 h. Smad7, PKR, p-eIF2*α* (Ser-51) and eIF2*α* were assessed in extracts of DLD-1 cells by western blotting. *β*-Actin was used as a loading control. Right panel: Quantitative analysis of p-eIF2*α* (Ser-51)/eIF2*α* protein ratio in total extracts of DLD-1 cells as measured by densitometry scanning of western blots. Values are expressed in arbitrary units (a.u.) and indicate the mean±S.E.M. of three experiments. (**d** and **e**) Representative histograms showing the percentage of DLD1 cells expressing ATF4 (**d**) and CHOP (**e**). Cells were transfected with either Smad7 S or AS oligonucleotide (both used at 2 *μ*g/ml). After 24 h, cells were incubated with either PKR-siRNA or scrambled-siRNA (both used at 100 nM) for further 24 h and then washed with PBS and cultured for additional 12 h. Data are presented as mean values of positive cells per high power field (h.p.f.)±S.E.M. of three independent experiments, in which at least two sections per group were analyzed

**Figure 3:**
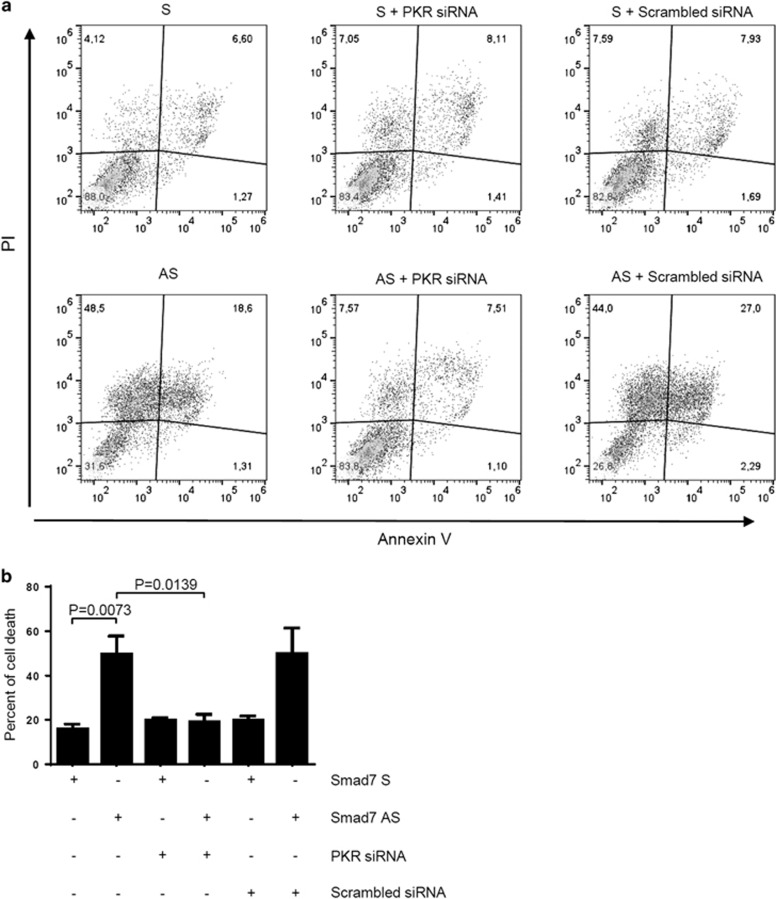
SMAD7 antisense (AS)-mediated DLD-1 cell death is reverted by PKR silencing. (**a**) Representative dot plots showing the percentages of AV- and/or PI-positive DLD-1 cells. Cells were transfected with either Smad7 sense (S) or AS oligonucleotide (both used at 2 *μ*g/ml). After 24 h, cells were incubated with either PKR-siRNA or scrambled-siRNA (both used at 100 nM) for further 24 h, and then washed with PBS and cultured for additional 48 h. (**b**) Representative histograms showing the percentage of cell death, as assessed by flow cytometry analysis of AV- and/or PI-positive cells, in DLD-1 cells treated as indicated in (**a**). Data are expressed as mean±S.E.M. of four experiments. *P*-values not significant are omitted

In neoplastic cells, phosphorylation of eIF2*α* can be promoted by three serine–threonine kinases including PKR,^9^ GCN2^10^ and PERK.^11^ To investigate whether these kinases were involved in the Smad7 antisense-induced eIF2*α* phosphorylation, we analyzed the phosphorylated/activated and total forms of each kinase in total proteins extracted from DLD-1 and HCT-116 cells treated with Smad7 sense or antisense. Inhibition of Smad7 led to the activation of PKR leaving unchanged the activation of GCN2 and PERK (Figure 2a and Supplementary Figure 2a). To examine whether PKR is involved in the Smad7 antisense-induced eIF2*α* phosphorylation, cells were treated with Smad7 antisense or sense and then exposed to PKR small interfering RNA (siRNA). Silencing of PKR abrogated Smad7 antisense-induced eIF2*α* phosphorylation (Figures 2b, c and Supplementary Figure 2b) and induction of ATF4 and CHOP (Figures 2d, e and Supplementary Figures 3). Block of CRC cells in the S phase in response to Smad7 antisense treatment is followed by enhanced cell death.^6^ Therefore, we next performed Annexin V (AV)/propidium iodide (PI) staining of cells treated as above to determine whether silencing of PKR and consequent inactivation of eIF2α abrogated Smad7 antisense-driven cell death. Data in Figure 3 show that the percentage of AV and/or PI-positive cells significantly increased following 72 h exposure to Smad7 antisense in comparison with that seen in cells treated with Smad7 sense and this phenomenon was reverted by PKR silencing.

These results suggest that Smad7 knockdown activates a PKR/eIF2α-dependent program that eventually leads to CRC cell death.

### Smad7 antisense-driven PKR activation is not secondary to PKR homodimerization, caspase activation, PACT induction, endoplasmic reticulum stress and unfolded protein response

**Figure 4:**
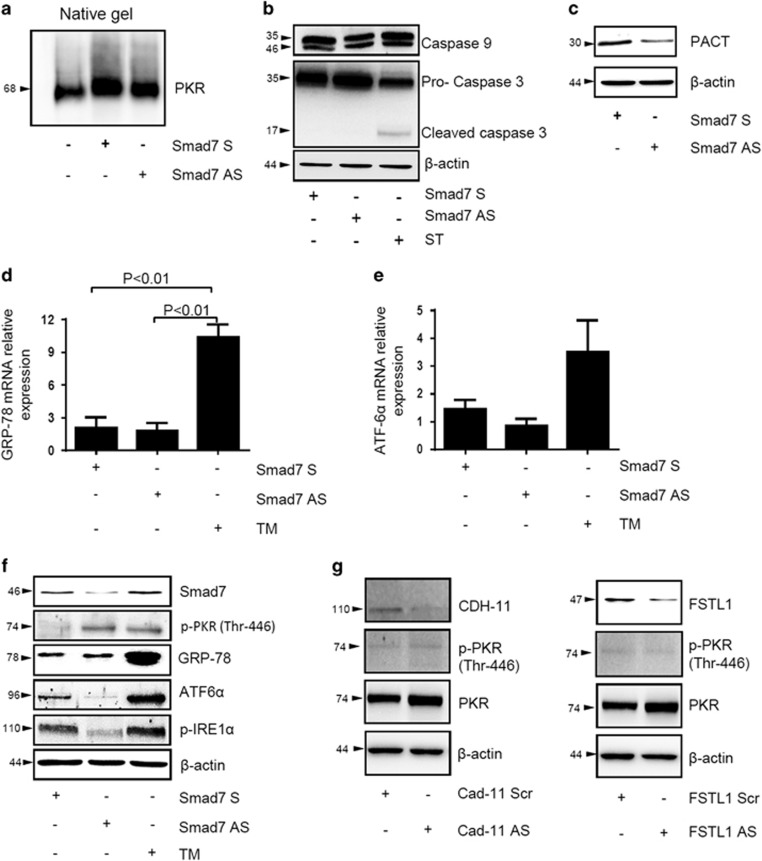
SMAD7 antisense (AS)-induced PKR phosphorylation in DLD-1 cells does not rely on the modulation of known PKR-activating pathways. (**a–c**) DLD-1 cells were transfected with either Smad7 sense (S) or AS oligonucleotide (both used at 2 *μ*g/ml). After 24 h, PKR (**a**), caspase-9, procaspase-3 and cleaved caspase-3 (**b**) as well as PACT expression (**c**) was assessed by western blotting. Staurosporine (ST) (1 *μ*g/ml) was used as a positive control for caspase-3 activation. *β*-Actin was used as loading control. One of three representative experiments is shown. (**d** and **e**) DLD-1 cells were transfected with either Smad7 S or AS oligonucleotide (both used at 2 *μ*g/ml). After 24 h, RNA transcripts for the ER stress-related genes *GRP-78* (**d**) and *ATF6α* (**e**) were determined by quantitative PCR. TM (1 *μ*g/ml) was used as a positive control. Levels are normalized to *β*-actin. Values mean±S.E.M. of three independent experiments. (**f**) DLD-1 cells were transfected with either Smad7 S or AS oligonucleotide (both used at 2 *μ*g/ml). After 24 h, Smad7, p-PKR (Thr-446), GRP-78, ATF6*α* and p-IRE1*α* expression was evaluated by western blotting. *β*-Actin was used as a loading control. (**g**) DLD-1 cells were transfected with either CAD-11 AS oligonucleotide (used at 400 nM) or FSTL1 AS oligonucleotide (used at 10 nM) along with the respective negative controls (scrambled). After 24 h, cells were washed with PBS and cultured for further 30 min. CDH-11, FSTL1, p-PKR (Thr-446) and PKR expression was assessed by western blotting. *β*-Actin was used as a loading control. One of three representative experiments is shown

Next, we evaluated mechanism(s) by which PKR is activated following Smad7 knockdown. Autophosphorylation of PKR in the activation segment containing Thr-446 and Thr-451 residues can be the result of PKR homodimerization.^12, 13^ To explore this possibility, total proteins were extracted from DLD-1 cells treated with Smad7 antisense or sense and separated on non-denaturing polyacrylamide gel electrophoresis. Next, membranes were incubated with a commercial antibody recognizing PKR. A single band of ~68 kDa, corresponding to full-length PKR, was seen in cells treated either with Smad7 antisense or sense, suggesting that activation of PKR in response to Smad7 knockdown is not secondary to PKR homodimerization (Figure 4a). PKR is a direct substrate of caspases and therefore its activation could be due to caspase activity. To address this issue, cells were transfected with Smad7 antisense or sense and expression of initiating caspase-9 and effector caspase-3 were evaluated after 24 h by western blotting. This time point was selected because our previous studies showed no induction of cell death after 24 h treatment with Smad7 antisense. Cells treated with Smad7 antisense exhibited no difference in terms of caspase-9 and procaspase-3 (Figure 4b). Moreover, no band corresponding to active form of caspase-3 was seen in the same cells (Figure 4b), arguing against the possibility that Smad7 antisense-induced PKR activation is mediated by caspases. A further factor controlling PKR activation is PKR-activating protein (PACT), a protein that directly binds and activates PKR in the absence of dsRNA.^14^ In DLD-1 cells, Smad7 inhibition associated with reduction, rather than increase, of PACT expression (Figure 4c).

Activation of the unfolded protein response (UPR) via endoplasmic reticulum (ER) stress has a pivotal role in the control of cell death. For this purpose, Smad7 antisense- or sense-transfected DLD-1 and HCT-116 cells were examined for the expression of the UPR marker, glucose-regulated protein-78 (GRP-78) and the UPR signaling pathway initiator ATF6*α* (Figures 4d, e, Supplementary Figures 5a and b). Figure 4f and Supplementary Figure 5c show no induction of such proteins following Smad7 knockdown. In the same studies, treatment of cells with tunicamycin (TM), an inducer of ER stress, increased expression of GRP-78, ATF6*α* and phosphorylated (p)-inositol-requiring enzyme-1*α* (IRE1*α*), another UPR signalling pathway initiator.

As PKR is activated by double-stranded RNAs, we next determined whether activation of PKR in Smad7 antisense-treated cells was indirectly due to cell transfection of antisenses. To address this issue, DLD-1 and HCT-116 cells were transfected with two additional antisenses against human cadherin-11 (CAD-11) or follistatin-related protein-1 (FSTL1). Transfection of cells with such oligonucleotides reduced expression of target proteins without changing the activation status of PKR (Figure 4g and Supplementary Figure 5d).

### Smad7 interacts with both p58^IPK^ and PKR, thereby promoting their interaction

**Figure 5:**
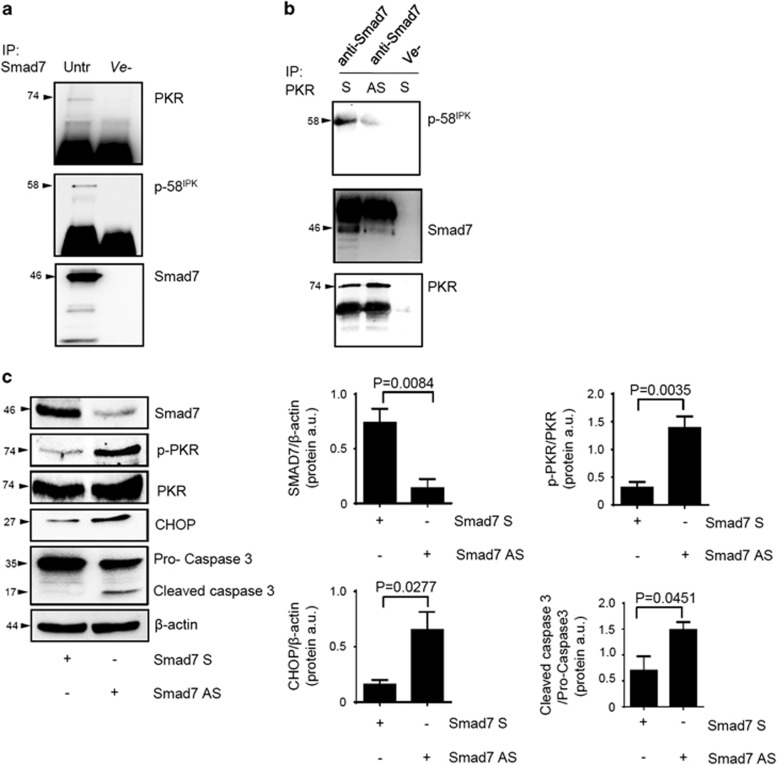
Smad7 knockdown prevents PKR-p-58^IPK^ interaction in DLD-1 cells and activates the PKR/CHOP axis in human CRC explants. (**a**) Total proteins extracted from DLD-1 cells were immunoprecipitated by an anti-human Smad7 or control isotype (ve−) antibody and then subjected to immunoblotting analysis using PKR, p58^IPK^ and Smad7 antibodies. (**b**) Total proteins extracted from DLD-1 cells transfected with either Smad7 sense (S) or antisense (AS) oligonucleotide (both used at 2 *μ*g/ml) were immunoprecipitated by an anti-human PKR or control isotype (ve−) antibody and then subjected to immunoblotting analysis using p58^IPK^, Smad7 and PKR antibodies. One of three representative experiments is shown. (**c**) Freshly obtained CRC explants were cultured in the presence of Smad7 S or AS oligonucleotide (both used at 8 *μ*g/ml) for 24 h. Smad7, p-PKR, PKR, CHOP, procaspase-3 and cleaved caspase-3 protein expression were assessed by western blotting. One of four representative experiments is shown. Right panels: Quantitative analysis of Smad7/*β*-actin, p-PKR/PKR, CHOP/*β*-actin and cleaved caspase-3/procaspase-3 protein ratio in extracts of CRC explants measured by densitometry scanning of western blots. Values are expressed in a.u. (arbitrary units) and are the means±S.E.M. of four experiments

p58^IPK^ is a molecular chaperone that regulates protein homeostasis.^15^ P58^IPK^ efficiently complexes with and inhibits PKR phosphorylation.^16^ Since Smad7 can interact with multiple proteins,^17^ we explored the possibility that in CRC cells Smad7 physically interacts with PKR and p58^IPK^, thereby facilitating interaction of these two proteins and consequent p58^IPK^-mediated PKR inhibition. By immunoprecipitation and immunoblotting of proteins extracted from DLD-1 and HCT-116 cells, we initially showed that Smad7 interacts with both PKR and p58^IPK^ (Figure 5a and Supplementary Figure 6). Importantly, knockdown of Smad7 reduces interaction of p58^IPK^ with PKR in DLD-1 cells (Figure 5b).

To confirm the above data, we used a well-established *ex vivo* organ system in which human CRC explants were incubated with Smad7 antisense or sense oligonucleotide. Inhibition of Smad7 enhanced p-PKR and CHOP expression and this effect was associated with a marked increase of cleaved caspase-3 (active form), a marker of apoptotic cells (Figure 5c).

## Discussion

The development of CRC is a multistage process characterized by a complex interaction between environmental carcinogens and genetic alterations and enhanced activation of various intracellular signals, which ultimately promote the expression of molecules involved in cell survival/antiapoptosis and cell cycle progression.^18^ We have previously shown that human and mouse CRC cells overexpress Smad7 and inhibition of such a protein suppresses neoplastic cell proliferation, a process that is characterized by enhanced phosphorylation of eIF2*α* and consequent arrest of cells in the S phase of the cell cycle.^6^ This study was undertaken to investigate the basic mechanisms by which Smad7 knockdown activates eIF2*α*. Initially we confirmed and expanded on our previous data showing that Smad7 and eIF2*α* colocalize in CRC cells and inhibition of Smad7 directly enhances eIF2*α* phosphorylation. eIF2*α* activation can be promoted by a family of four well-characterized serine–threonine kinases, which curtail general translation in response to many cellular stresses.^8, 10, 11^ These kinases include PERK, PKR, GCN2 and heme-regulated eIF2*α* kinase, even though heme-regulated eIF2*α* kinase function seems to be restricted to erythroid cells.^19^ Our data strongly support the involvement of PKR in the eIF2*α* phosphorylation seen in Smad7-deficient cells. Indeed, knockdown of Smad7 but neither CAD-11 nor FSTL1 selectively activates PKR. Moreover, Smad7 inhibition has no effect on PERK and GCN2. Finally, we show that the ability of Smad7 antisense oligonucleotide to induce eIF2*α* phosphorylation is greatly reduced in CRC cells after PKR silencing by siRNA. PKR silencing also prevents the Smad7 antisense oligonucleotide-driven CRC cell death in line with the role of this kinase in the control of cell survival/death.^20^ Analysis of events downstream of PKR activation that mediate cell death indicates that PKR-dependent apoptosis is associated with Fas-associated death domain-mediated activation of caspase 8 and upregulation of Fas and Bax.^21, 22^ Interestingly, PKR regulates cellular apoptosis not only through eIF2*α* phosphorylation, but also through modulation of other factors such as STAT, IRF1, p53, JNK and p38, as well as engaging the NF-kB pathway.^23^ Therefore, we cannot exclude the possibility that abrogation of the Smad7 antisense oligonucleotide-induced cell death seen in cells treated with PKR-siRNA is due to the modulation of other pathways other than eIF2*α*. The demonstration that Smad7 antisense oligonucleotide activates PKR and interferes with intracellular pathways that sustain CRC cell survival is consistent with previous studies showing that the activity against CRC of other anti-neoplastic drugs (e.g. non-steroidal anti-inflammatory drug) relies in part on PKR activation.^24^ PKR is activated by several distinct stress signals or cellular factors.^25, 26^ Therefore, we next evaluated the factors/mechanisms involved in PKR activation following transfection of CRC cells with Smad7 antisense oligonucleotide. By western blotting, we show that activation of PKR in Smad7-deficient cells is not the result of PKR homodimerization, early action of PKR-activating caspases or changes in the expression of PACT, a protein that interacts with and activates PKR.^14^ Endogenous imbalances in cells, such as that secondary to overproduction of proteins, accumulation of mutant proteins or loss of calcium homeostasis, can cause a malfunction of cellular processes and stress to the ER system.^27^ In response to ER stress, UPR program is activated and serves to minimize the accumulation and aggregation of misfolded proteins by increasing the capacity of the ER machinery to fold proteins correctly and activate the degradation of aberrant proteins.^28^ Under normal conditions, the master regulator of the UPR, GRP-78, is associated with stress sensor proteins in the ER luminal domain. Under stress conditions, GRP-78 is released and binds to misfolded proteins, including ATF6*α* and IRE1*α*.^29^ No increase in GRP-78, ATF6*α* and p-IRE1*α* is seen in Smad7-deficient cells arguing against the possibility that activation of PKR is secondary to UPR program activation. Studies in various cell systems have shown that PKR can be negatively regulated by p58^IPK^, a member of the tetripeptide repeat family of proteins that regulates PKR even in the absence of virus infection.^30^ There is also evidence that p58^IPK^ may function as an oncogene and that one mechanism by which the protein induces malignant transformation is through the downregulation of PKR activity and subsequent deregulation of protein synthesis.^31^ Since Smad7 interacts with multiple intracellular proteins, we explored the possibility that in CRC cells Smad7 can interact with both p58^IPK^ and PKR, thus facilitating the inhibitory effect of p58^IPK^ on PKR activation. Immunopreciptation and immunoblotting of extracts prepared from unstimulated CRC cells show interaction of Smad7 with the two proteins. Knockdown of Smad7 reduces interaction of p58^IPK^ with PKR, thus suggesting a putative mechanism by which Smad7 deficiency associates with enhanced PKR activation.

In conclusion, our data show that inhibition of Smad7 activates in CRC cells a program of cell death that is mediated by activation of PKR and support further the notion that Smad7 is a valid target for therapeutic strategies in CRC.

## Materials and Methods

### Inhibition of Smad7 by Smad7 antisense oligonucleotide

Custom LNA oligonucleotide targeting Smad7 was provided by Exiqon (Vedbaek, Denmark). Smad7 sense or Smad7 antisense oligonucleotide was used at the final concentration of 2 *μ*g/ml.

### Cell culture

All reagents were from Sigma-Aldrich (Milan, Italy) unless specified. The human CRC cell lines HCT-116 and DLD-1 were obtained from the American Type Culture Collection (ATCC, Manassas, VA, USA). Cells were maintained in McCoy's 5A (HCT-116) and RPMI-1640 (DLD-1) medium, all supplemented with 10% fetal bovine serum (FBS), 1% penicillin/streptomycin (P/S) (all from Lonza, Verviers, Belgium) in a 37 °C, 5% CO_2_, fully humidified incubator. Cell lines were recently authenticated by STR DNA fingerprinting using the PowerPlex 18D System Kit (Promega, Milan, Italy) according to the manufacturer's instructions. The STR profiles of all the cell lines matched the known DNA fingerprints.

To investigate the mechanisms by which Smad7 inhibition leads to eIF2a hyperphosphorylation, HCT-116 and DLD-1 cells were transfected with either Smad7 antisense or Smad7 sense oligonucleotide (both used at 2 *μ*g/ml) for 24 h using Opti-MEM medium and Lipofectamine 3000 reagent (both from Life Technologies, Milan, Italy) according to the manufacturer's instructions. Cells were then washed with phosphate-buffered saline (PBS) and recultured with the respective fresh medium containing 0.05% bovine serum albumin (BSA) for further 5 min until 24 h depending on experiment settings. The efficiency of the transfection was determined by western blotting analysis.

To evaluate whether Smad7 regulates eIF2*α* through PKR, cells were transfected with PKR-siRNA or scrambled-siRNA (both used at 100 nM; Santa Cruz Biotechnology, Santa Cruz, CA, USA) for 24 h using Opti-MEM medium and Lipofectamine 3000 reagent as described above.

In some experiments, cells were treated with 1 *μ*g/ml TM (Sigma-Aldrich) as a positive control for ER stress.

### Patients, samples and organ culture

Tissue samples were taken from the tumoral area of patients who underwent colon resection for sporadic CRC at the Tor Vergata University Hospital (Rome, Italy) and used for organ culture experiments. All patients received neither radiotherapy nor chemotherapy before undergoing surgery. The human studies were approved by the local Ethics committee and each patient gave written informed consent. CRC explants were placed on Millicell inserts (EMD Millipore, Milan, Italy) in 6-well plate containing RPMI-1640 medium supplemented with 10% FBS, 1% P/S and 50 *μ*g/ml gentamycin in the presence of either Smad7 antisense oligonucleotide or Smad7 sense oligonucleotide (both used at 8 *μ*g/ml) for 24 h. The culture was performed in an organ culture chamber at 37 °C in a 5% CO_2_/95% O_2_ atmosphere.

### Cytospin preparation of cultured cells

DLD1 and HCT-116 cell lines were harvested by trypsinization and washed with either RPMI-1640 or McCoy's 5A medium respectively. Aliquots of 15 000 cells were fixed with 70% ethanol and centrifuged at 230 r.c.f. for 5 min on glass slides. Cytospins were dried and stored at room temperature before use.

### Fluorescence studies

For confocal studies, cells were plated on a 20 mm glass coverslip at a density of 20 000 cell per cm^2^. After 24 h, cells were washed in PBS and then fixed for 10 min with 4% formaldeyde. Afterwards, they were washed in PBS and permeabilized for 10 min with 0.1% Triton X-100 at room temperature (RT). Nonspecific binding was reduced by incubating cells with 5% BSA at RT for 1 h. Cells were then incubated with anti-Smad7 antibody diluted 1:50 in PBS (R&D Systems, Minneapolis, MN, USA) and with anti-eIF2*α* antibody diluted 1:50 in PBS (Santa Cruz Biotechnology) for 16 h at 4 °C. Cells were then washed three times with PBS and then incubated for 60 min with Alexa Fluor 488F(ab)2 goat anti-mouse (Molecular Probes, Thermo Fisher Scientific, Waltham, MA, USA) or with Alexa Fluor 568 F(ab)2 goat anti-rabbit diluted 1:1000 in 5% BSA. Cells were then rinsed three times with PBS and finally nuclei were stained for 10 min using 1 mg/ml DAPI (Molecular Probes) diluted in PBS. After a final wash, cells were mounted using Prolong Antifade Kit (Molecular Probes) and observed through a Nikon ECLIPSE Ti confocal microscope (Nikon Instruments Inc., Melville, NY, USA). Images were acquired using NIS Elements AR 4.00.04 software (Nikon, Nikon Instruments Inc.).

In fixed cells, antigen retrieval was performed at high temperature and cells were stained with primary antibodies followed with secondary antibodies. Primary antibodies were as follows: anti-Smad7 (1:50 final dilution; R&D Systems), anti-eIF2*α*, anti-CHOP (respectively, used at 1:50 and 1:100 final dilution; Santa Cruz Biotechnology), anti-p-eIF2*α* and anti-ATF4 (both used at 1:100 final dilution; Cell Signaling Technology, Danvers, MA, USA). The corresponding anti-mouse and anti-rabbit secondary antibodies were labeled with Alexa Fluor 488 (green staining) or Alexa Fluor 568 (red staining, both from Invitrogen Thermo Fisher Scientific, Waltham, MA, USA). In each separate immunofluorescence experiment, negative controls prepared by omitting the respective primary antibody were included. Cytospins were counterstained with 4,6-diamidino-2-phenylindole (DAPI) (Life Technologies) and evaluated using a fluorescence microscope (Olympus, Milan, Italy).

### Inhibition of CAD-11 and FSTL1 by LNA oligonucleotides

Custom LNA oligonucleotides targeting CAD-11 and FSTL1 were provided by Exiqon. HCT-116 and DLD-1 cells were transfected for 24 h with either CAD-11 or FSTL1 antisense, used at the final concentration of 400 and 10 nM, respectively, using Opti-MEM medium and Lipofectamine 3000 reagent (both from Life Technologies) according to the manufacturer's instructions. Control A oligonucleotide was used as a negative control of the transfection.

### Immunoprecipitation and western blotting

Cell lines were lysed on ice in buffer containing 10 mM HEPES (pH 7.9), 10 mM KCl, 0.1 mM ethylenediaminetetraacetic acid, 0.2 mM ethylene glycol-bis(*β*-aminoethyl ether)-*N*,*N*,*N*',*N*'-tetraacetic acid and 0.5% Nonidet P40 supplemented with 1 mM dithiothreitol, 10 mg/ml aprotinin, 10 mg/ml leupeptin, 1 mM phenylmethylsulfonyl fluoride, 1 mM Na_3_VO_4_ and 1 mM NaF. Lysates were clarified by centrifugation at 4 °C, 12 000 r.c.f. for 30 min and separated on sodium dodecyl sulfate (SDS)-polyacrylamide gel electrophoresis. Blots were incubated with the following antibodies: ATF6*α*, caspase-9, CHOP, GRP-78, eIF2*α*, PERK and p-PERK (Thr-981) (1:500 final dilution; all from Santa Cruz Biotechnology), cleaved caspase-3, GCN2, p-eIF2*α* Ser-51, p58^IPK^, PACT and PKR (1:1000 final dilution; all from Cell Signaling Technology), p-IRE1*α* and p-GCN2 (Thr-899) (used at 1:1000 and 1:500 final dilution, respectively, both from Abcam, Cambridge, UK ), p-PKR (Thr-446) (1:500 final dilution; Biorbyt, San Francisco, CA, USA), CAD-11 and FSTL1 (1:500 and 1:1000 final dilution, respectively; both from Thermo Scientific, Rockford, IL, USA), Smad7 (0.5 *μ*g/ml; R&D Systems), procaspase-3 (1:1000 final dilution; Millipore-Upstate, Lake Placid, NY, USA) followed by a secondary antibody conjugated to horseradish peroxidase (used at 1:20 000 final dilution; Dako Agilent Technologies, Glostrup, Denmark). After analysis, each blot was stripped and incubated with either a mouse anti-human ERK1 antibody (1:500 final dilution; Santa Cruz Biotechnology) or mouse anti-human *β*-actin antibody (final dilution 1:5000; Sigma-Aldrich) to ascertain equivalent loading of the lanes. Computer-assisted scanning densitometry (Chemidoc Touch Images; Bio-Rad, Hercules, CA, USA) was used to analyze the intensity of the immunoreactive bands.

For native polyacrylamide gel electrophoresis, all reagents were used without SDS and proteins were run at 4 °C.

For immunoprecipitation, protein lysates were preincubated with 2 *μ*g of anti-Smad7 antibody (Santa Cruz Biotechnology) or anti-PKR (1:100 final dilution; Abcam), separated by SDS-polyacrylamide gel electrophoresis, and immunoblotted with antibodies against Smad7 (0.5 *μ*g/ml; R&D Systems), eIF2*α* (1:500 final dilution; Santa Cruz Biotechnology), p58^IPK^ and PKR (both used at 1:1000 final dilution; Cell Signaling Technology). Computer-assisted scanning densitometry (Chemidoc Touch Images; Bio-Rad) was used to analyze the intensity of the immunoreactive bands.

### Analysis and quantification of cell death

To score cell death, cells were transfected with either Smad7 antisense oligonucleotide or Smad7 sense oligonucleotide (both used at 2 *μ*g/ml) in the presence or absence of PKR/scrambled-siRNA (both used at 100 nM). After 24 h, cells were washed with PBS and recultured with fresh medium containing 0.05% BSA for further 48 h. Cells were then collected, washed two times in AV buffer, stained with fluorescein isothiocyanate–AV (1:100 final dilution; Immunotools, Friesoyte, Germany) according to the manufacturer's instructions and incubated with 5 mg/ml of PI for 30 min at 4 °C, and their fluorescence was measured using FL-1 and FL-3 channels of Gallios Flow Cytometer (Beckman Coulter, Life Sciences, Pasadena, CA, USA). Analysis was performed using the FlowJo software (FlowJo LLC, Ashland, OR, USA).

### RNA extraction, complementary DNA preparation and real-time PCR

Total RNA was extracted from cells using TRIzol reagent (Life Technologies) according to the manufacturer's instructions . A constant amount of RNA (1 *μ*g per sample) was retrotranscribed into complementary DNA, and 1 *μ*l of complementary DNA per sample was then amplified using the following conditions: denaturation 1 min at 95 °C, annealing 30 s at 56 °C for GRP-78 and ATF6*α* or 30 s at 60 °C for *β*-actin, followed by 30 s of extension at 72 °C. Primers used were as follows: GRP-78 – forward, 5′-GGTGAAAGACCCCTGACAAA-3′ and reverse, 5′-GTCAGGCGATTCTGGTCATT-3′ ATF6 – forward, 5′-TCAGGGAGTGAGCTACAAGT-3′ and reverse, 5′-CTTGTGGTCTTGTTATGGGT-3′.

Real-time PCR was performed using the IQ SYBR Green Supermix (Bio-Rad) and RNA expression was calculated relative to the housekeeping *β*-actin gene on the base of the ΔΔCt algorithm.

### Statistical analysis

Values are expressed as mean±S.E.M. and results analyzed using the two-tailed Student's *t*-test or the one-way analysis of variance followed by Bonferroni's *post hoc* test for multiple comparisons. GraphPad Prims6 (GraphPad Software, La Jolla, CA, USA) was used for statistical and graphical data evaluations. Significance was defined as *P*-values <0.05.
