# Supplementary material for: Smad7 knockdown activates protein kinase RNA-associated eIF2α pathway leading to colon cancer cell death
Source: Cell Death Dis. 2017 Mar 16;8(3):e2681–. doi: 10.1038/cddis.2017.103 (PMC5386514; doi:10.1038/cddis.2017.103)
Supplement: Supplementary Information [file cddis2017103x1.docx]

**Supplementary Figure Legends**

**Supplementary Figure 1. Smad7 co-localizes with eIF2α in colon cancer cells and controls eIF2α downstream signaling**. **(a)** Representative confocal laser scanning microscopy images showing Smad7 and eIF2α co-localization in HCT-116 cell line. The scale bars are 25 μm; the scale bar in the inset is 10 μm. **(b)** Total proteins extracted from HCT-116 cells were immunoprecipitated by an anti-human Smad7 or control isotype (ve-) antibody and then subjected to immunoblotting analysis using eIF2α and Smad7 antibodies. One of three representative experiments in which similar results were obtained is shown. **(c)** Representative immunofluorescence pictures of HCT-116 cells showing that Smad7 knockdown enhances eIF2α (Ser-51) phosphorylation. Cells were transfected with either Smad7 sense (S) or antisense (AS) oligonucleotide (both used at 2μg/ml). After 24 hours, cells were cultured for further 6 hours, fixed and stained as described in (a). One of three representative experiments is shown. The scale bars are 20 μm. (**d-e)**  Representative immunofluorescence images of HCT-116 cells showing an increase of ATF4 and CHOP expression following Smad7 knockdown. Cells were transfected with either Smad7 S or AS (both used at 2μg/ml). After 24 hours, cells were cultured for further 12 hours, fixed and stained with DAPI nuclear staining (blue), anti-ATF4 or anti-CHOP and secondary Alexa Fluor 546 antibody (red). One of three representative experiments is shown. The scale bars are 20 μm.

**Supplementary Figure 2. Smad7 AS-induced eIF2α phosphorylation in HCT-116 cells relies on PKR activation. (a)** HCT-116 cells were transfected with either Smad7 sense (S) or antisense (AS) oligonucleotide (both used at 2μg/ml). After 24 hours, cells were washed with PBS and cultured for 5, 15 and 30 minutes. P-PERK (Thr-981), p-GCN2 (Thr-899) and p-PKR (Thr-446) expression was assessed by Western blotting. One of three representative experiments is shown. **(b)** Left panel. Cells were transfected with either Smad7 S or AS oligonucleotide (both used at 2μg/ml). After 24 hours cells were incubated with either PKR-siRNA or scrambled-siRNA (both used at 100nM) for further 24 hours and then washed with PBS and cultured for additional 6 hours. Smad7, PKR, p-eIF2α (Ser-51), eIF2α were assessed in extracts of HCT-116 cells by Western blotting. β-actin was used as loading control. Right panel. Quantitative analysis of p-eIF2α (Ser-51)/eIF2α protein ratio in total extracts of HCT-116 cells as measured by densitometry scanning of Western blots. Values are expressed in arbitrary units (a.u.) and indicate the mean ± SEM of three experiments.

**Supplementary Figure 3. PKR knockdown reduces ATF4 and CHOP expression in Smad7 deficient cells.** Representative immunofluorescence images of DLD-1 cells show a decrease of ATF4 (left panel) and CHOP (right panel) expression following PKR knockdown in Smad7-deficient cells. Cells were transfected with either Smad7 S or AS oligonucleotide (both used at 2μg/ml). After 24 hours cells were incubated with either PKR-siRNA or scrambled-siRNA (both used at 100nM) for further 24 hours and then washed with PBS and cultured for additional 12 hours. Finally, cells were fixed and stained with DAPI nuclear staining (blue), anti-ATF4 or anti-CHOP and secondary Alexa Fluor 546 antibody (red). One of three representative experiments is shown. The scale bars are 20 μm.

**Supplementary Figure 4. PKR knockdown reduces ATF4 and CHOP expression in Smad7 deficient cells. (a)** Representative immunofluorescence images of HCT-116 cells show a decrease of ATF4 (left panel) and CHOP (right panel) expression following PKR knockdown in Smad7-deficient cells. Cells were transfected with either Smad7 S or AS oligonucleotide (both used at 2μg/ml). After 24 hours cells were incubated with either PKR-siRNA or scrambled-siRNA (both used at 100nM) for further 24 hours and then washed with PBS and cultured for additional 12 hours. Finally, cells were fixed and stained with DAPI nuclear staining (blue), anti-ATF4 or anti-CHOP and secondary Alexa Fluor 546 antibody (red). One of three representative experiments is shown. The scale bars are 20 μm. **(b-c)** Representative histograms show the percentage of HCT-116 cells expressing ATF4 (b) and CHOP (c). Data are presented as mean values of positive cells per high power field (hpf) ± SEM of three independent experiments in which at least two sections per group were analyzed.

**Supplementary Figure 5. SMAD7 AS-induced PKR phosphorylation in HCT-116 cells does not rely on the modulation of known PKR-activating pathways. (a-b)** HCT-116 cells were transfected with either Smad7 sense (S) or antisense (AS) oligonucleotide (both used at 2μg/ml). After 24 hours, RNA transcripts for GRP-78 **(a)** and ATF6α **(b)** were determined by qPCR. Tunicamycin (TM) (1μg/ml) was used as positive control. Levels are normalized to β-actin. Values mean ± SEM of three independent experiments. **(c)** HCT-116 cells were transfected with either Smad7 S or AS oligonucleotide (both used at 2μg/ml). After 24 hours, Smad7, p-PKR (Thr-446), GRP-78, ATF6α and p-IRE1α expression was evaluated by Western blotting. β-actin was used as loading control. **(d)** HCT-116 cells were transfected with either cadherin 11 antisense (CAD-11 AS) oligonucleotide (used at 400nM) or FSTL1 AS oligonucleotide (used at 10nM) along with the respective negative controls (scrambled). After 24 hours, cells were washed with PBS and cultured for further 30 minutes. CDH-11, FSTL1, p-PKR (Thr-446) and PKR expression was assessed by Western blotting. β-actin was used as loading control. One of three representative experiments is shown.

**Supplementary Figure 6. Smad7 interacts with PKR and p-58^IPK^ in HCT-116 cells.** Total proteins extracted from HCT-116 cells were immunoprecipitated by an anti-human Smad7 or control isotype (ve-) antibody and then subjected to immunoblotting analysis using PKR, p58^-IPK^ and Smad7 antibodies.
