# Supplementary figures and images for: Smad7 knockdown activates protein kinase RNA-associated eIF2α pathway leading to colon cancer cell death
Source: Cell Death Dis. 2017 Mar 16;8(3):e2681–. doi: 10.1038/cddis.2017.103 (PMC5386514; doi:10.1038/cddis.2017.103)

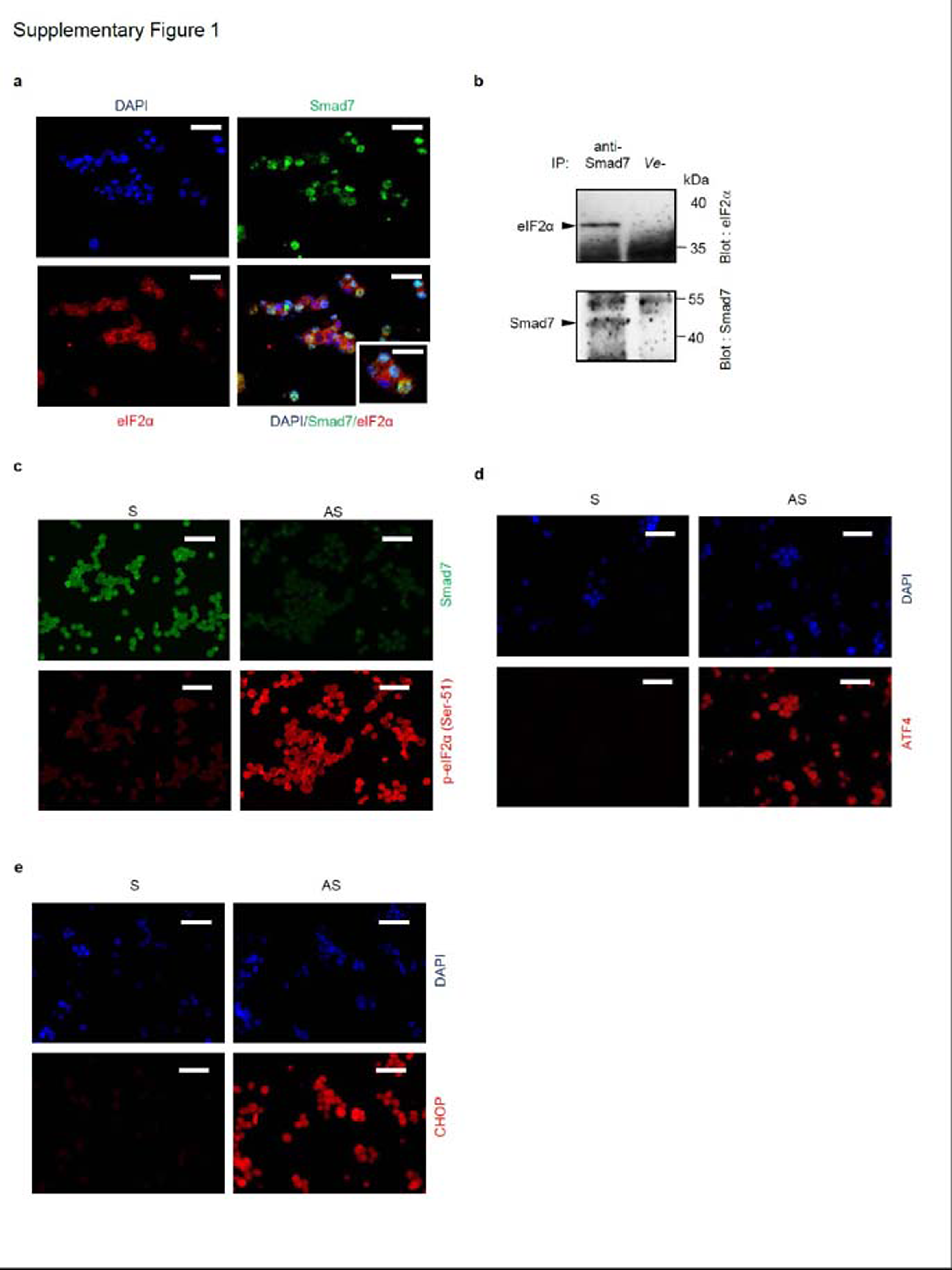

Supplement: Supplementary Figure 1 [file cddis2017103x2.tif]

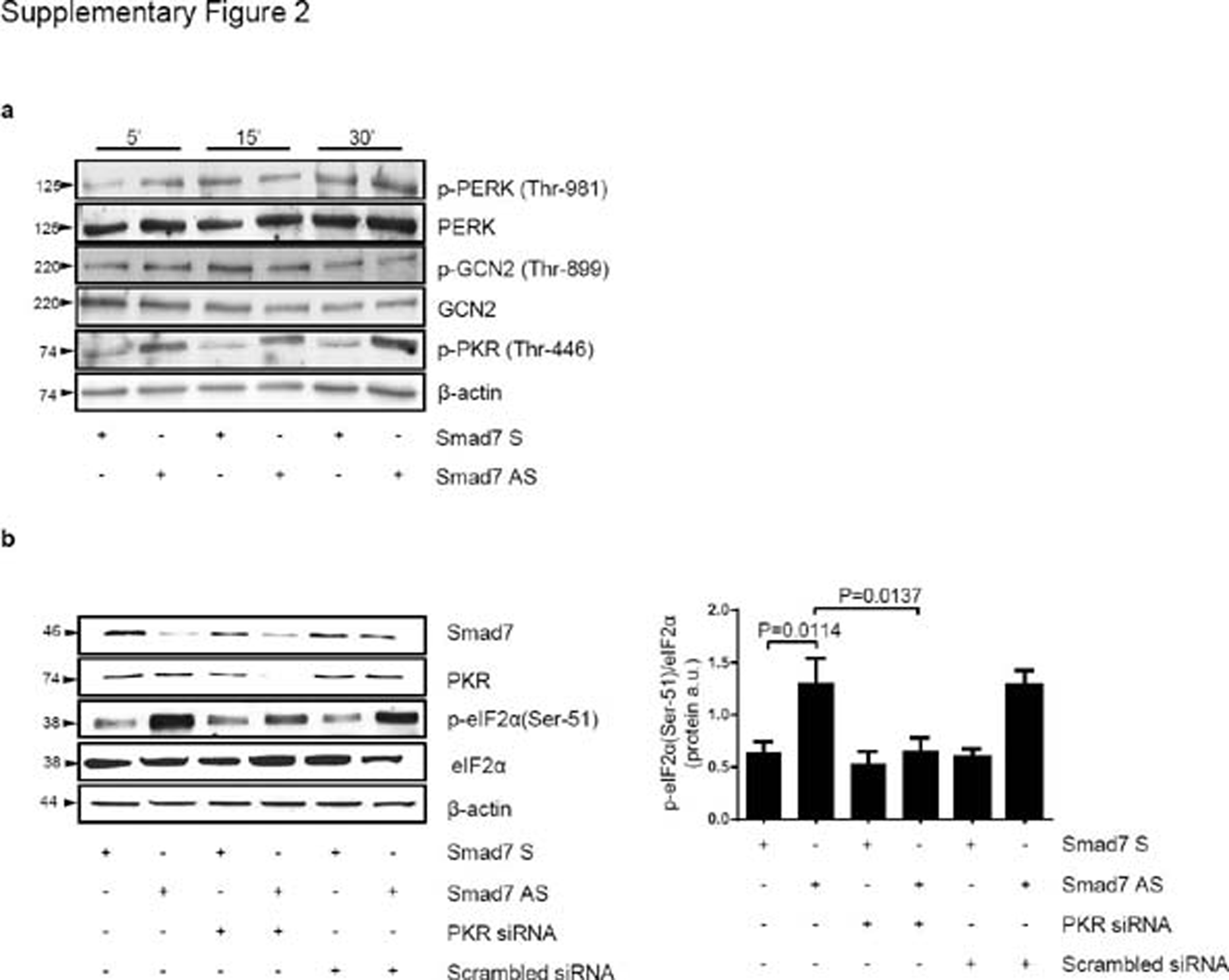

Supplement: Supplementary Figure 2 [file cddis2017103x3.tif]

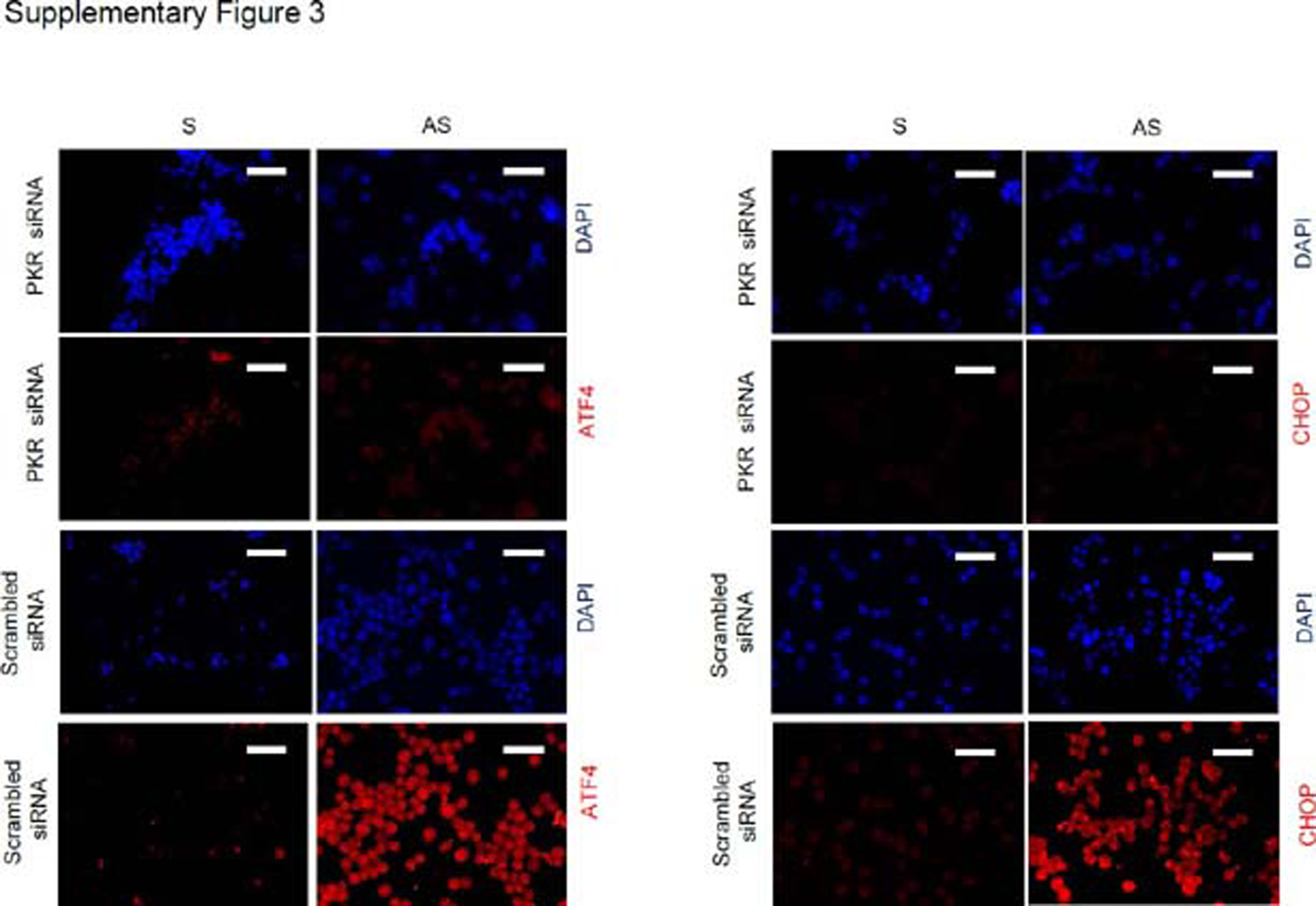

Supplement: Supplementary Figure 3 [file cddis2017103x4.tif]

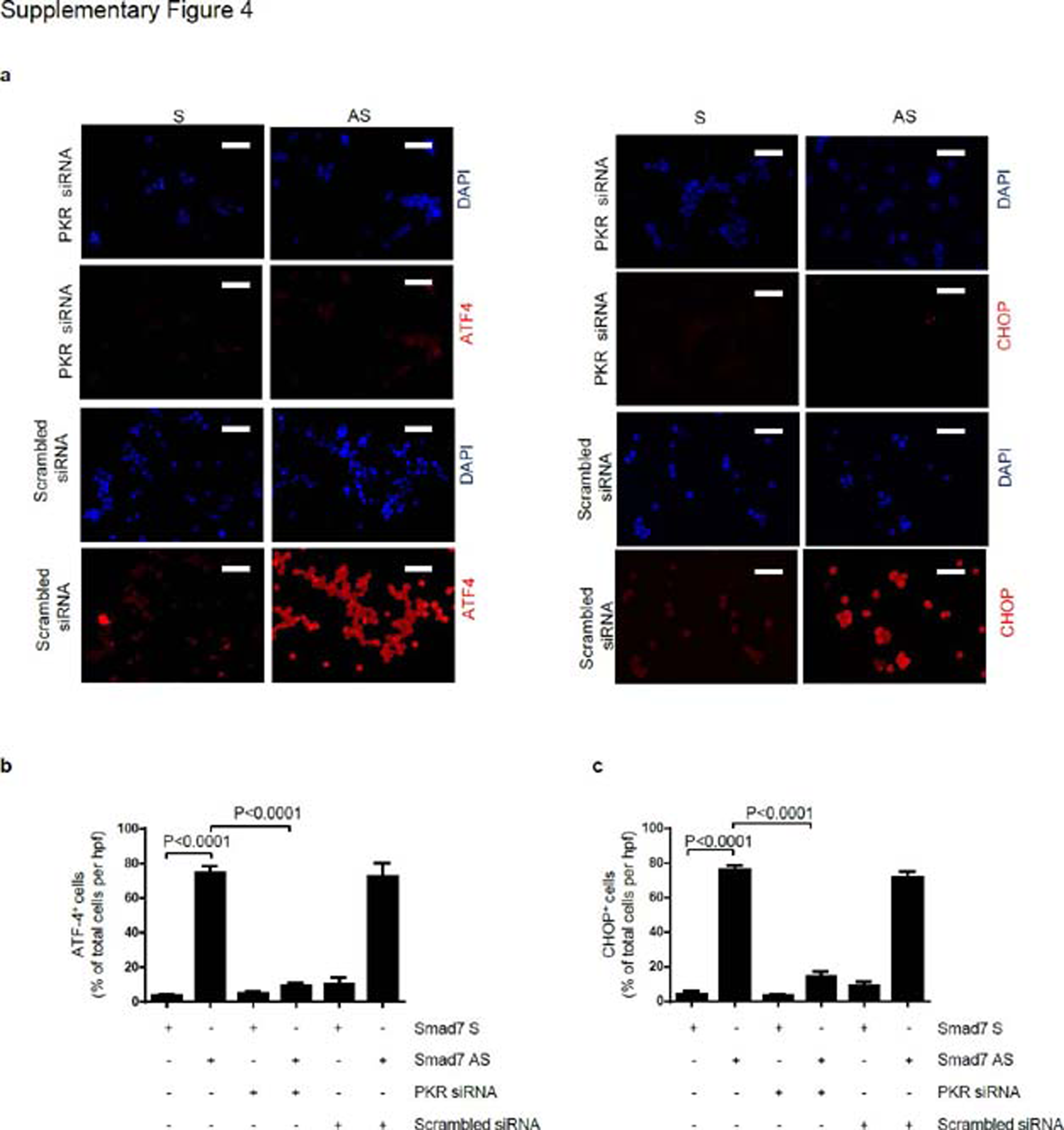

Supplement: Supplementary Figure 4 [file cddis2017103x5.tif]

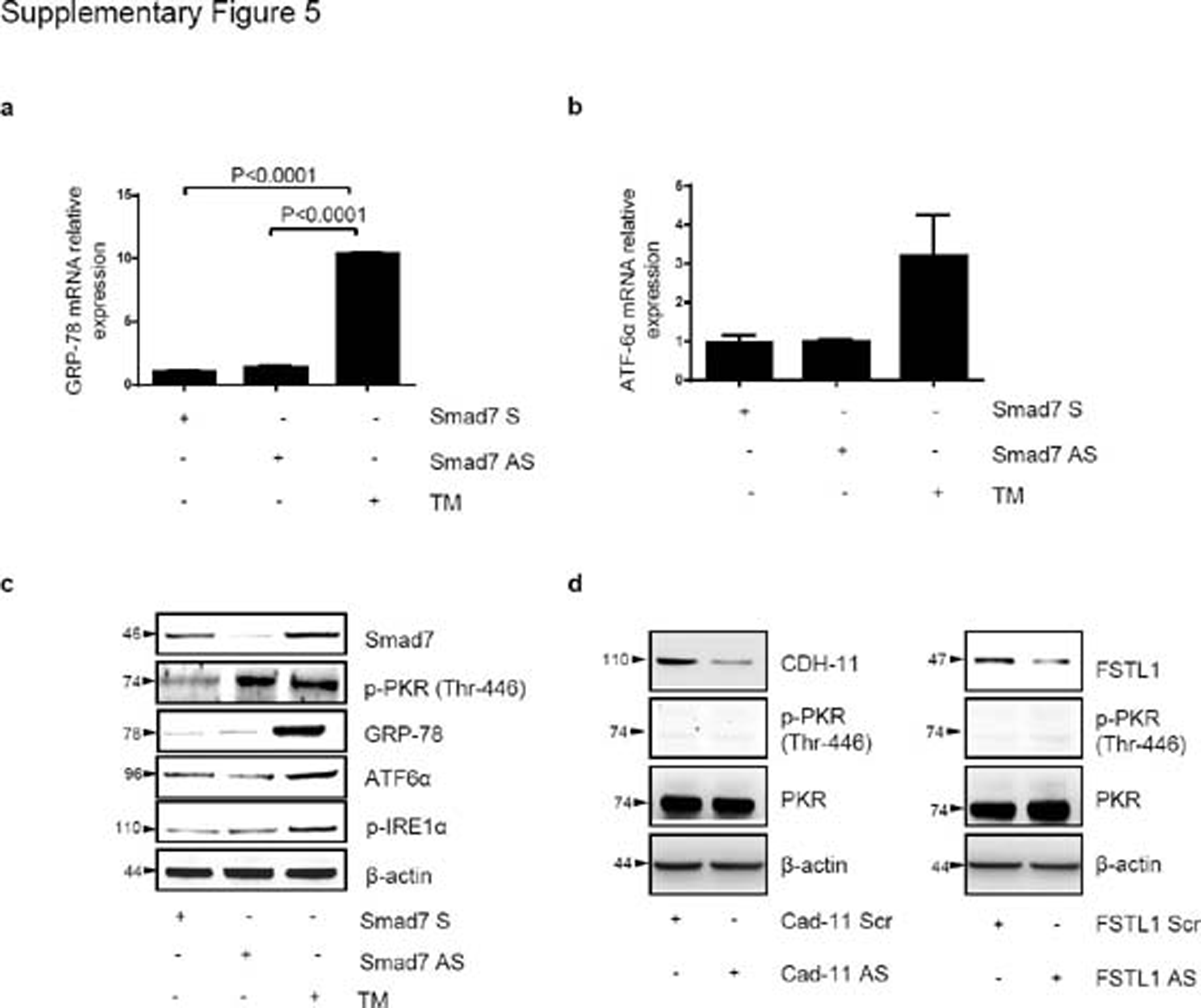

Supplement: Supplementary Figure 5 [file cddis2017103x6.tif]

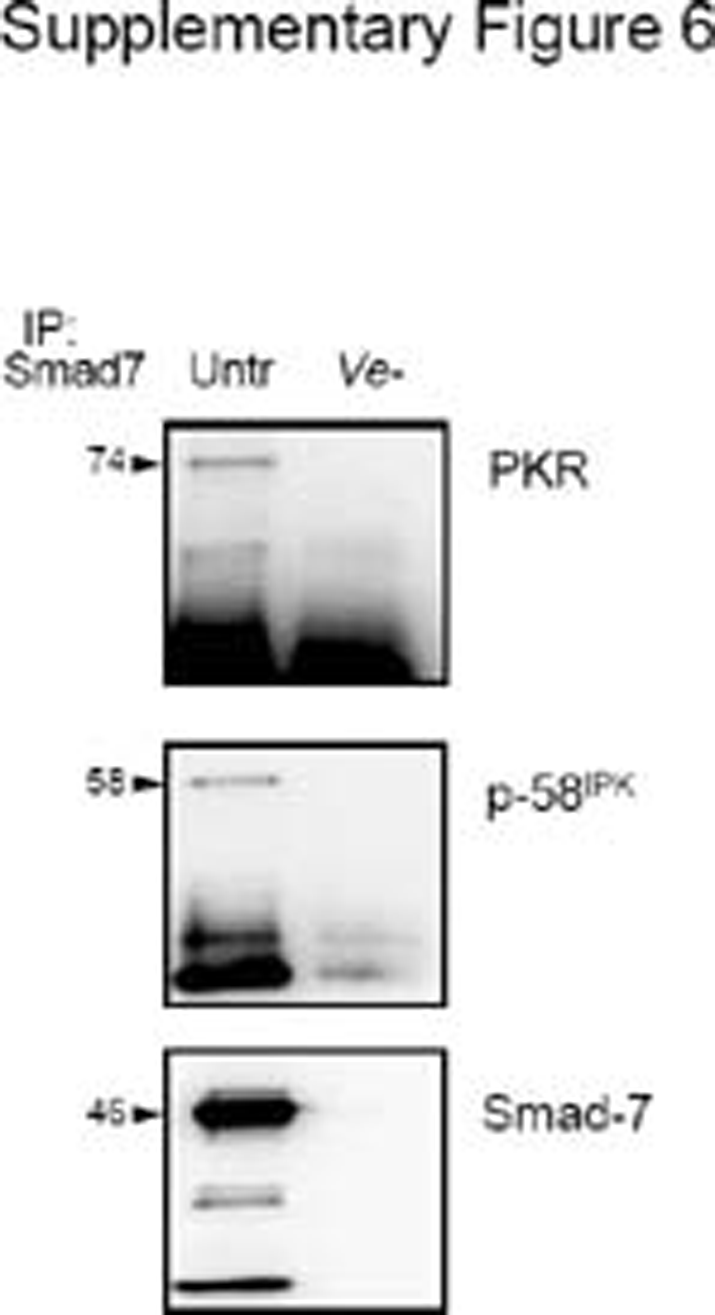

Supplement: Supplementary Figure 6 [file cddis2017103x7.tif]
